# Supplementary figures and images for: A probabilistic, distributed, recursive mechanism for decision-making in the brain
Source: PLoS Comput Biol. 2018 Apr 3;14(4):e1006033. doi: 10.1371/journal.pcbi.1006033 (PMC5882111; doi:10.1371/journal.pcbi.1006033)

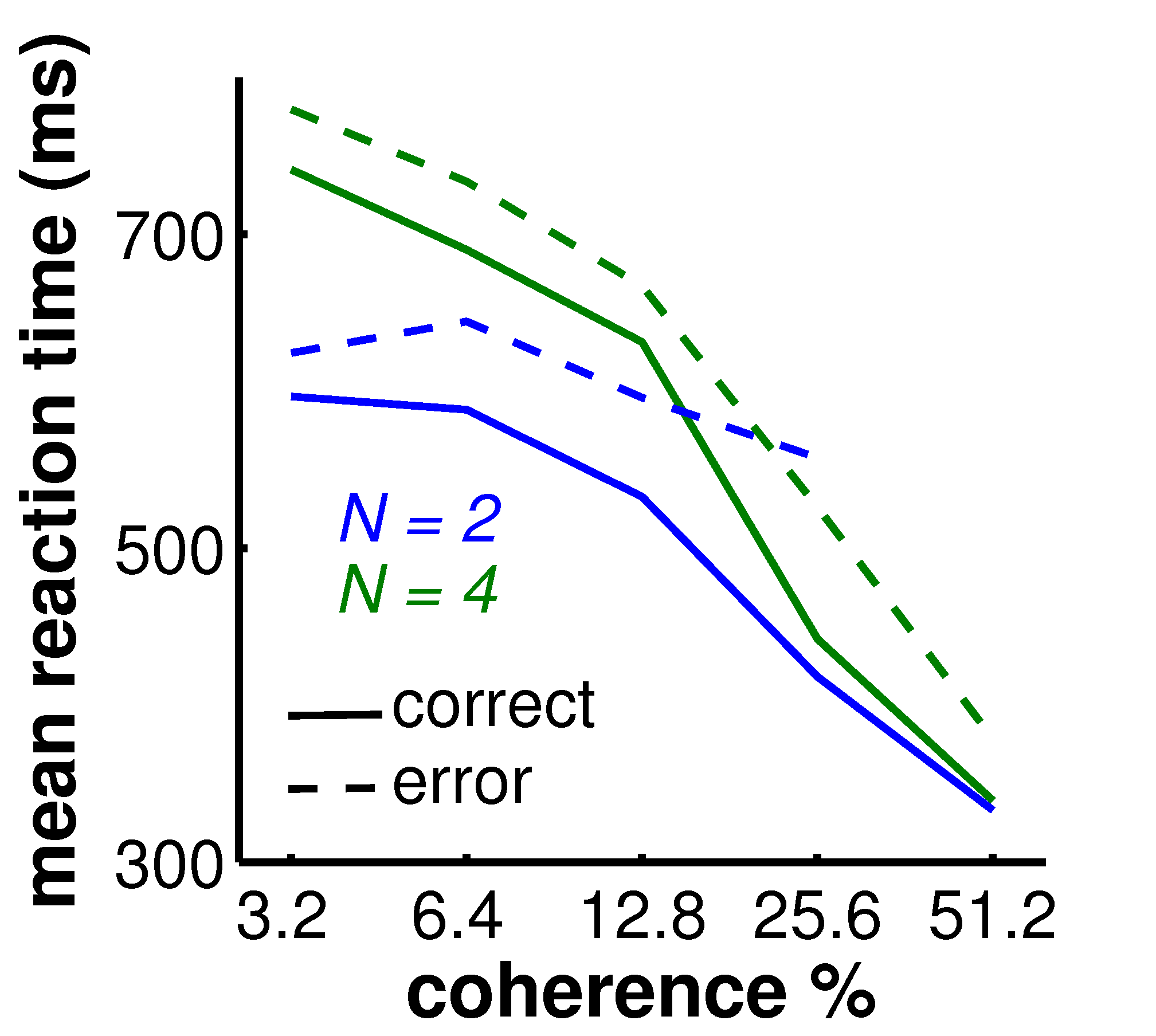

Supplement: S1 Fig — Monkey mean reaction times from Fig 4, panels a and b, shown in the same plot for comparison. Solid: correct trials. Dashed: error trials. Blue, green: N = 2, 4. (TIF) [file pcbi.1006033.s001.tif]

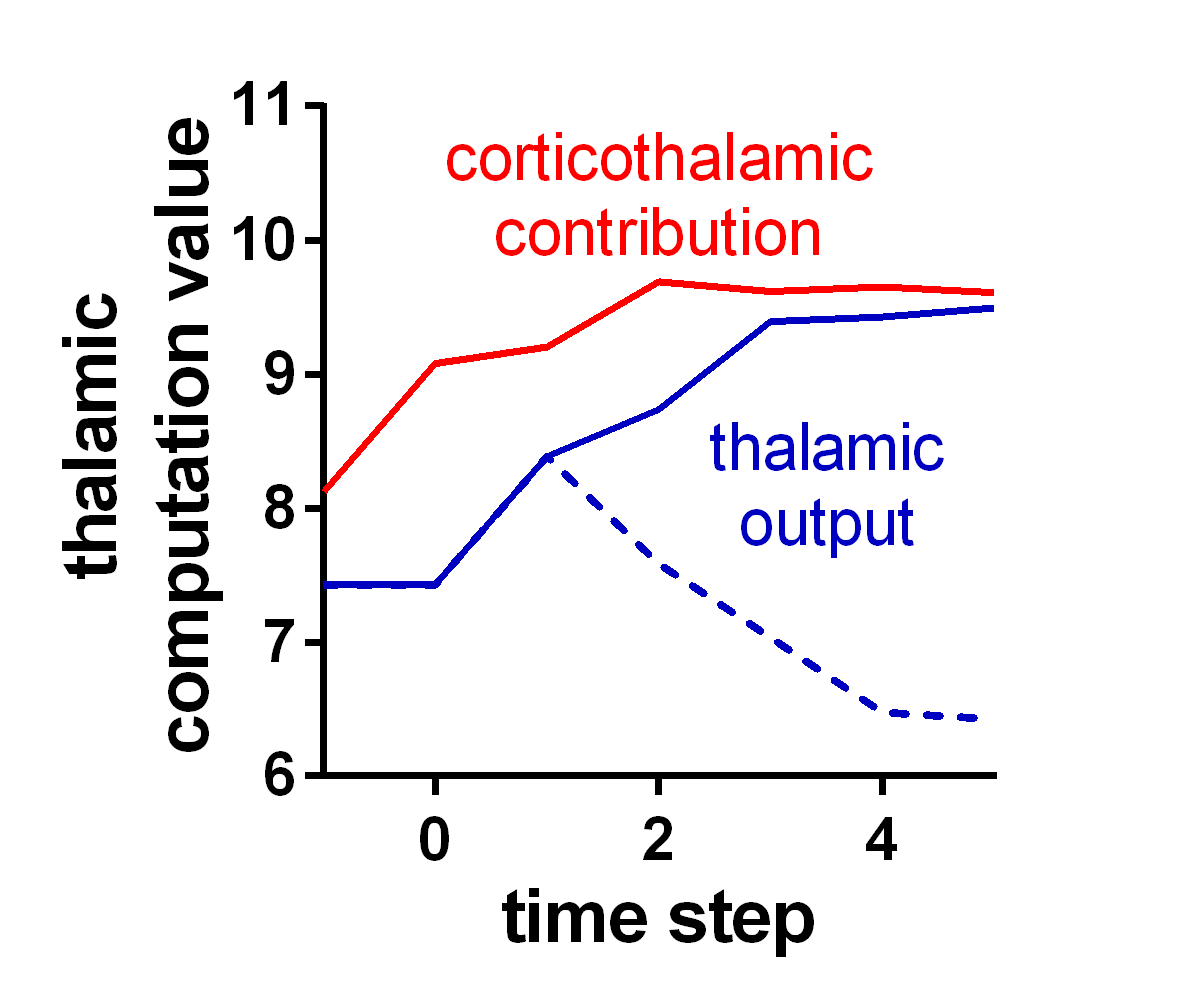

Supplement: S2 Fig — Example mean cortico-thalamic contribution, h(t − δbu) (red), compared to the mean thalamic output during inRF settings (solid blue) and outRF ones (dashed blue) for 25% coherence and N = 2. Single Monte Carlo experiment with 800 total trials. (TIF) [file pcbi.1006033.s002.tif]

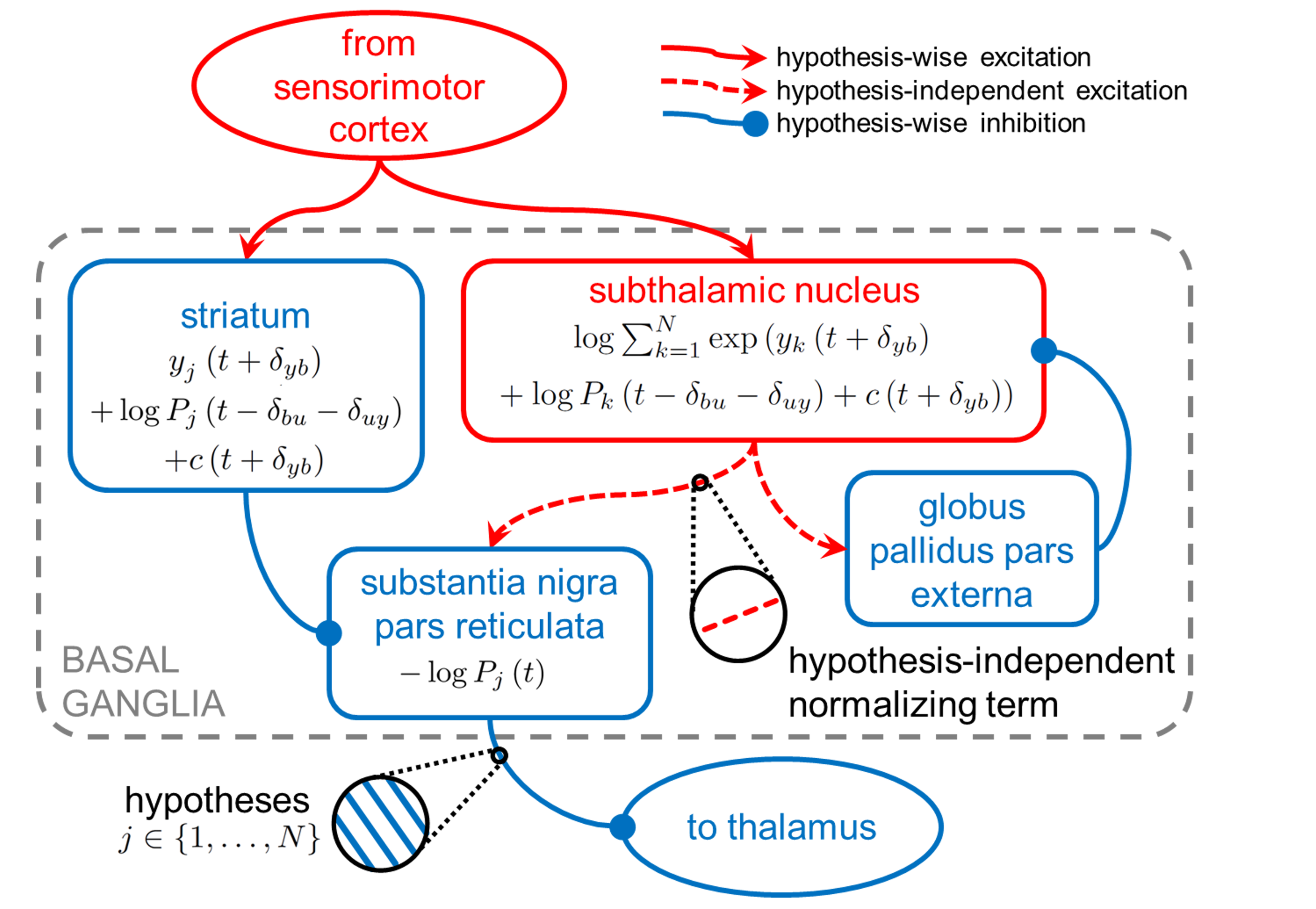

Supplement: S3 Fig — Parallel computations for N hypotheses—indexed by j—mapped onto the basal ganglia nuclei, within the grey dashed box (see [17, 18, 62]). This may include the pathway from striatum to the globus pallidus pars externa, as well as the pathway from the latter to substantia nigra pars reticulata, as demonstrated by [17], thus representing all major pathways among the basal ganglia. It has recently been shown that neurons in the microcircuitry joining the rodent subthalamic nucleus and globus pallidus, are theoretically able to collectively represent the normalization term required by algorithms in the family of the (r)MSPRT [21]; this, as well showing that the mapping of MSPRT (thus rMSPRT) to the basal ganglia can also account for a connection from globus pallidus pars externa to striatum. Same conventions and notation as in Fig 5. All computations are delayed with respect to the substantia nigra pars reticulata. log∑k=1Nexp(yk(t+δyb)+logPk(t-δbu-δuy)+c(t+δyb)): normalization term (from Eq 9), putting together the cortical computations for all hypotheses into a hypothesis-independent contribution. Note that the model striatum represents a copy of the cortical signal (as in Fig 5) but its influence on the substantia nigra pars reticulata is the negative of such cortical input. (TIF) [file pcbi.1006033.s003.tif]

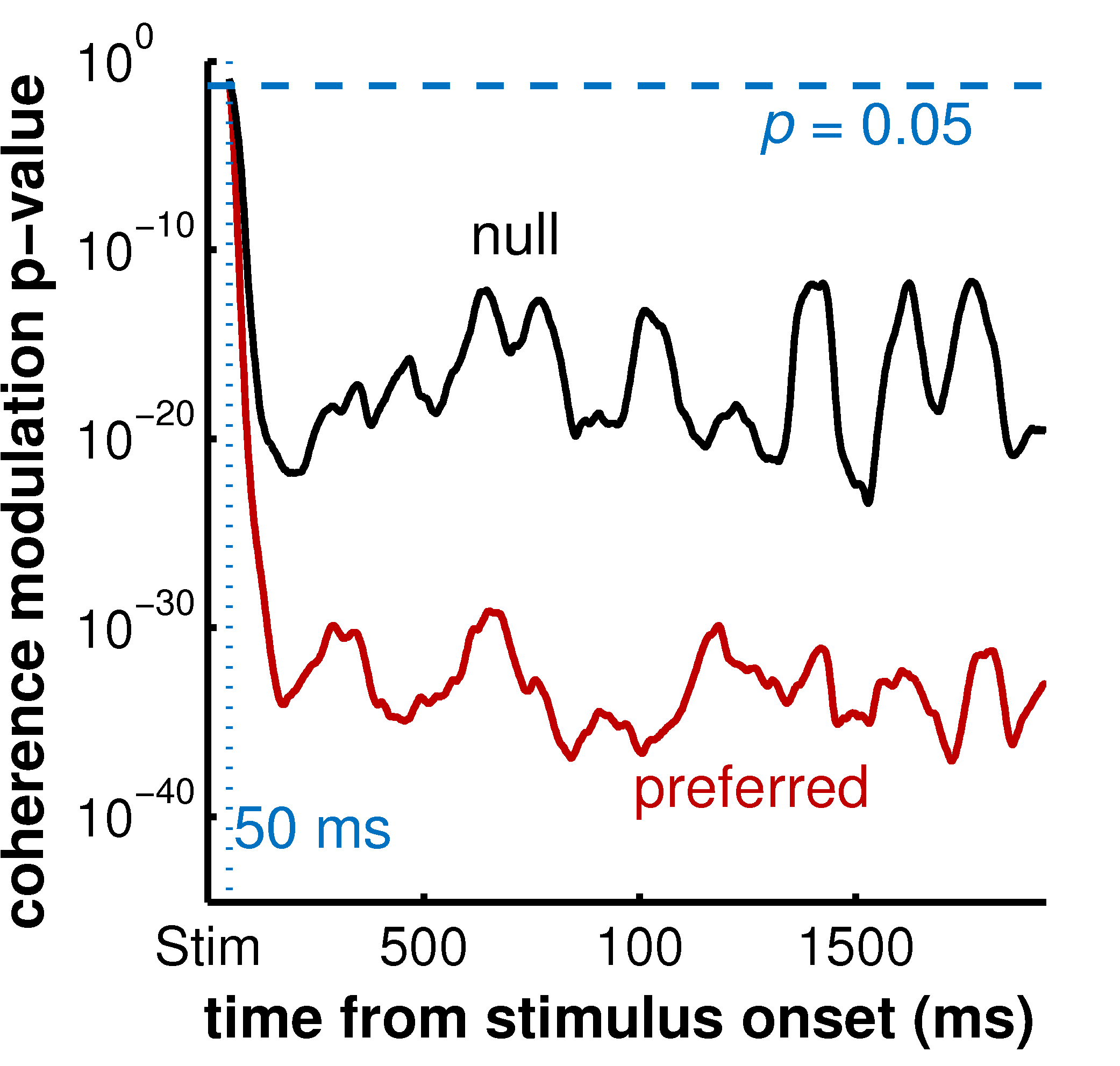

Supplement: S4 Fig — With the method described in the main article, we computed the mean firing rate of every MT neuron in our data facing dots moving in its preferred or null motion directions at six coherence levels (%): 0, 3.2, 6.4, 12.8, 25.6, 51.2 (randomly assigning 0% trials between directions). For every neuron, every 1 ms bin, we conducted a linear regression per motion direction of the form v = β + βss, where v are the mean firing rates at every bin, s are the corresponding coherence levels (%), and β, βs are the intercept and the coefficient for the coherence contribution, respectively. We then applied a t—test where the null hypothesis was: the mean of the distribution of the 189–213 βs’s (again, one per MT neuron) we got per direction, equals 0. Here we show the corresponding p—value for MT coherence modulation, per direction, aligned at stimulus onset (Stim). Note that this involved conducting a single statistical test/comparison for every 1 ms bin, independent of those conducted in surrounding bins. Red: dots moving in the preferred direction of recorded neuron. Black: moving in the opposite, null direction. Horizontal blue dashed line: p = 0.05. Vertical blue dotted line: 50 ms. The coherence modulation p—values for preferred and null directions drop under 0.05 about ∼ 50 ms after the onset of the dots stimulus and drop much further soon after this. (TIF) [file pcbi.1006033.s004.tif]
